# Study on TiO_2_ Nanofilm That Reduces the Heat Production of Titanium Alloy Implant in Microwave Irradiation and Does Not Affect Fracture Healing

**DOI:** 10.1155/2022/4910731

**Published:** 2022-04-13

**Authors:** Yiming Xu, Zikai Hua, Yun Cai, Xianxuan Feng, Jiajia Yang, Jie Shen, Yuehong Bai

**Affiliations:** ^1^Department of Rehabilitation Medicine, Shanghai Jiao Tong University Affiliated Sixth People's Hospital, 200233, China; ^2^Orthotek Laboratory, School of Mechatronics Engineering and Automation, Shanghai University, 200072, China; ^3^Fudan University Affiliated Zhongshan Hospital, 200032, China; ^4^The First Affiliated Hospital, Sun Yat-sen University, Guangzhou, 510062, China; ^5^Medical Records and Statistics Office, Shanghai Jiao Tong University Affiliated Sixth People's Hospital, 200233, China

## Abstract

**Background:**

Metal implants can produce heat and damage adjacent tissues under microwave irradiation, which makes local metal implants in the body a contraindication for microwave therapy. However, with the wide application of titanium alloy implants which have low permeability and low conductivity, this concept has been challenged. Our team members have confirmed through previous research that continuous low-power microwave irradiation does not cause thermal damage to the surrounding tissues of the titanium alloy. Is there any other way to further increase the dose of microwave irradiation while reducing the heat production of titanium alloy implants? In this study, the effect of TiO_2_ nanofilm on reducing the heat production of titanium alloy implants in microwave field was verified by animal experiments, and the effect of TiO_2_ nanofilm on fracture healing was observed.

**Methods:**

30 rabbits were selected. In the experiment of temperature measurement, 10 rabbits were randomly divided into experimental group (*n* = 5) and control group (*n* = 5), and the contralateral lower limb of the rabbits in experimental group was set as the sham operation group. The right femurs in the experimental group were implanted with Ti6Al4V plates coated with TiO_2_ nanofilm, and the right femurs in the control group were implanted with common titanium alloy plates without TiO_2_ nanofilm. The same surgical procedure was used in the sham operation group, but no plate was implanted. The temperature of the deep tissue above the metal implant was measured with an anti-interference thermocouple thermometer during 20 minutes of microwave irradiation. The other 20 rabbits were randomly divided into two groups, experimental group (*n* = 10) and control group (*n* = 10). The femoral shaft fracture models were established again. Ti6Al4V plates coated with TiO_2_ nanofilm and common titanium alloy plates were implanted in the two groups, respectively, and both groups were exposed to continuous microwave irradiation with a power of 40 W or 60 W for 30 days after operation. The fracture healing was evaluated by X-ray at 0 day, 14 days, and 30 days after microwave irradiation, respectively. The animals were sacrificed at 30 days after operation for histopathological assessment.

**Results:**

The temperature in the experimental group, control group, and sham operation group increased significantly after 40 W and 60 W microwave irradiation (2.18 ± 0.15°C~6.02 ± 0.38°C). When exposed to 40 W microwave, the temperature rise of the control group was 4.0 ± 0.34°C, which was significantly higher than that of the experimental group 2.82 ± 0.15°C (*P* < 0.01) and the sham operation group 2.18 ± 0.33°C (*P* < 0.01). There was no significant difference in temperature rise between the experimental group and the sham operation group (*P* = 0.21). When exposed to 60 W microwave, the temperature rise of the control group was 6.02 ± 0.38°C, which was significantly higher than that of the experimental group 3.66 ± 0.14°C (*P* < 0.01) and sham operation group 2.96 ± 0.22°C (*P* < 0.01), and there was no significant difference between the experimental group and the sham operation group (*P* = 0.32). X-ray evaluation showed that there was no significant difference in callus maturity between the experimental group and the control group at 14 days (*P* = 0.554), but there was significant difference in callus maturity between the two groups at 30 days (*P* = 0.041). The analysis of bone histologic and histomorphometric data at 30 days was also consistent with this.

**Conclusion:**

Under the animal experimental condition, compared with the common titanium alloy implant, the TiO_2_ nanofilm can reduce the heat production of the titanium alloy implant in the 2450 MHz microwave field and has no adverse effect on fracture healing. This study opens up a promising new idea for the application of microwave therapy to metal implants in human body.

## 1. Introduction

Microwave is a high-frequency electromagnetic wave with wavelength from 1 mm to 1 m and frequency from 300 MHz to 300 GHz [1]. When the human body is exposed to a microwave irradiation, an induced electromagnetic field is generated in the body, which heats the tissues. It is well known that the therapeutic effect of microwave is mainly related to heat production, and microwave therapy is also known as microwave diathermy. Microwave diathermy can improve the range of motion of joints in clinical application [2]. Enhance microcirculation and protein synthesis to heal wounds [3], increase blood flow to injured tissues [4], reduce pain [5], and modify the physical properties of fibrous tissues [6]. Therefore, microwave therapy is widely used in the rehabilitation of acute injury and chronic inflammation of bone [7–9], joints [10], muscles, and tendons [11, 12].

The local metal implants in human body are generally considered to be an absolute contraindication for microwave therapy [13], which is mainly due to the secondary irradiation of tissues caused by the microwave reflection of metal and the excessive heat generated by the eddy current formed inside the metal implants in the microwave electromagnetic field, which damages the tissues adjacent to the metal implants [14, 15]. This contraindication limits the application of microwave in persistent pain, delayed healing, and infection after internal fixation [16–19].

This problem has existed since the application of microwave in clinic, and now clinicians and material engineering scientists are trying to solve it. Inspired by the fact that patients with titanium alloy implants can undergo MRI examination [20–22], our team believes that patients fixed with titanium alloy steel plate may receive microwave treatment because of the low permeability and low conductivity of titanium alloy. The following experiments verify this hypothesis. In vivo animal studies by Ye et al. [23, 24] showed that there was no obvious histological change in the skeletal muscles and nerves near the titanium alloy implant when single irradiation with 2450 MHz microwave at a lower dose (20 W~40 W) or continuous irradiation with 25 W titanium alloy fixed rabbit femoral fracture. However, with the increase of power (60 W and 80 W), acute thermal injury of skeletal muscle and nerve could be observed. The results of these studies indicate that, on the one hand, low doses microwave can be applied to fractures fixed by titanium alloy implants. On the other hand, the heat generated by microwave irradiation of titanium alloy implants is dose-dependent.

Is there any other way to further increase the microwave irradiation dose while reducing the heat generation of titanium alloy implants? Some materials scientists are attracted by surface modification and coating techniques, which have been applied to improve the performance of modern orthopaedic materials [25, 26]. Professor Hua's team from Shanghai University has designed a TiO_2_ nanofilm that was coated on titanium alloy (Ti6Al4V) substrates by sol-gel method. Preliminary study in vitro [27] showed that the temperature rise of the sample was lower than that of the uncoated control group after the sample was radiated by 2450 MHz 25 W microwave for 10 minutes, which met the expected requirements. In order to further verify the in vivo effect of the TiO_2_ nanofilm and observe the effect of the TiO_2_ nanofilm on fracture healing, we designed following animal experiments.

## 2. Materials and Methods

### 2.1. Sample Size

In a previous study with a power of 40 W microwave [24], the temperature change in the deep tissue above the implant in each group of subjects obeyed a normal distribution with a standard deviation of 0.42°C. If the true difference between the experimental and control means is 1.3°C, we would need to study three experimental samples and three control samples in order to reject the zero hypothesis that the thermal change of the experimental group and the control group is equal to the probability of 0.8(power). The type I error probability associated with this null hypothesis is 0.05. In this study, there were 5 measurements for each condition.

### 2.2. Animal Model

Thirty New Zealand white rabbits weighing 3.0~3.5 kg were used in the study. Ten of 30 rabbits were used for temperature measurement trial and were randomly divided into experimental group (EG) and control group (CG), with 5 rabbits in each group. The contralateral lower limb of the rabbits in experimental group was set as the sham operation group (SOG). 20 of 30 rabbits were used for bone histology and imaging observation and were divided into EG and CG, with 10 rabbits in each group. The animals were purchased from Songlian Laboratory Animal Corporation (production license: SCXK2017-0008).

**Figure 1:**
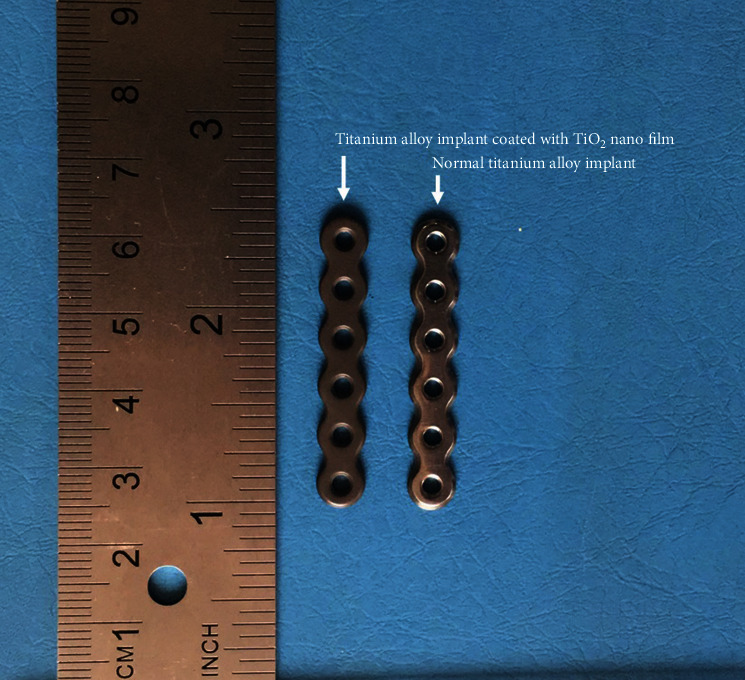
Plates used in the EG and the CG.

**Figure 2:**
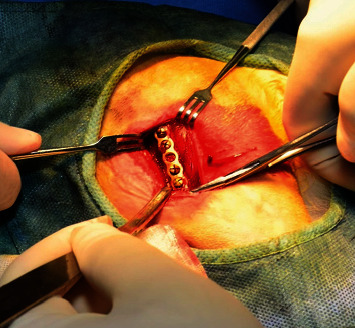
Implantation of titanium alloy plate into rabbit femur.

To establish an animal model of internal fixation of femoral shaft fracture in rabbits, the skin of the right lateral thigh was incised under intravenous anesthesia with pentobarbital sodium 30 mg/kg, and the fascia and skeletal muscle were separated layer by layer to the middle of the femur. The EG was implanted with a 460 mm × 42 mm “I”-shaped titanium alloy plate coated with TiO_2_ nanofilm (Figures 1 and 2) (Zhengtai Laboratory of Shanghai University, China). The CG was implanted with titanium alloy plate without TiO_2_ nanofilm (Synthes Company, USA). Then, a stainless steel wire saw was used to cut the middle femur corresponding to the fourth hole of the plate, with a cutting depth of about 3 mm. In the end, wash the wound, suture the wound layer by layer, and bandage it. In the SOG, only skin incision and muscle separation were performed on the left thigh to expose the femur, and no plate was implanted. Penicillin (800,000 units/day) was administered intramuscularly 3 days after surgery. At the end of the experiment, all New Zealand rabbits were sacrificed by excessive anesthesia (pentobarbital sodium).

### 2.3. Microwave Irradiation and Temperature Measurement

**Figure 3:**
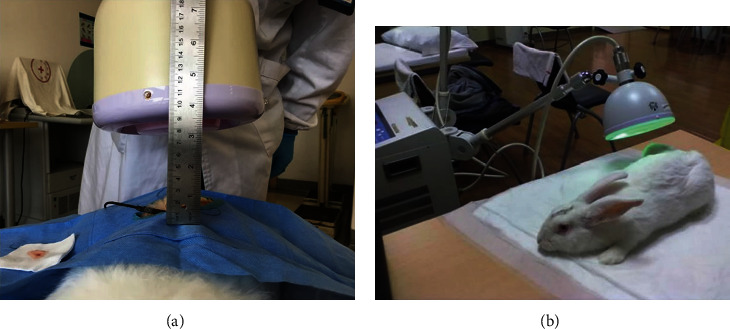
(a) A 2450 MHz microwave transmitting probe was placed 10 cm above the skin, and an anti-interference thermocouple thermometer sensor (indicated by the red arrow in the figure) was inserted through the original skin incision for temperature measurement. (b) The microwave transmitting probe was placed 10 cm above the surgical incision and faced the middle of thigh during daily microwave irradiation.

Three days after operation, the EG, the CG, and the SOG received the temperature measurement during microwave irradiation. The ambient temperature of the laboratory is set at 25°C. New Zealand rabbits were anesthetized with sodium pentobarbital (30 mg/kg) via the ear vein, and the original skin incision was exposed and cut. The temperature sensor of the anti-interference thermocouple thermometer (FHCME-04008, Baldwin, USA) was embedded in the deep tissue 5 mm above the central surface of the implant. Leave it for at least 3 minutes, and when the digital reading is stable, record the initial temperature. Then, a 2450 MHz microwave transmitting probe (PM-800, ITO, Japan) was placed 10 cm above the skin (Figure 3(a)), the microwave irradiation power was adjusted to 40 W, and the irradiation was started, and the irradiation time and the corresponding thermometer readings were recorded for 20 minutes. Then, the microwave therapeutic apparatus was turned off and waited for 1 hour. Observe the temperature displayed by the thermometer to recover to the initial temperature, turn on the microwave therapeutic apparatus again, adjust the microwave irradiation power to 60 W, and record the irradiation time and the corresponding thermometer reading for 20 minutes.

### 2.4. Continuous Microwave Irradiation

Twenty New Zealand rabbits in the EG and CG were exposed to 2450 MHz microwave irradiation every day from the third day after operation until histological and imaging assessment. The microwave-emitting probe was placed 10 cm above the incision in the thigh during daily irradiation (Figure 3(b)) at a power of 40 W for 20 minutes.

### 2.5. X-Ray Assessment

Anteroposterior and lateral radiographs of the femur were taken at 0, 14, and 30 days after microwave irradiation to evaluate the fracture healing process. The camera output voltage is 55 KV, the current is 0.3 mA, the exposure time is 3 s, and the optical density evaluation uses the same light source. The X-ray images were stored in the PC as digital image files. The X-ray images of each specimen were evaluated by three radiologists. The degree of fracture healing was based on the maturity of callus. The classification of Goldberg et al. is as follows [28]: stage 1, unhealed; stage 2, possible healing; and stage 3, radiographic healing, followed by calculation of the mean radiographic score.

### 2.6. Histology and Histomorphometery

After 30 days of microwave irradiation, 5 rabbits in each group were sacrificed, and 10 samples were taken for histological assessment. Specimens were fixed with 10% formalin and decalcified with 10% nitric acid. After embedding in paraffin, 5 *μ*m thick longitudinal sections were prepared and stained with hematoxylin-eosin or Masson trichrome. Two slides of each specimen were examined, one stained with hematoxylin-eosin and the other stained with Masson trichrome. A group of pathologists (LZW, JQ, and DJX) blinded to trial grouping scored all stained sections based on the amount of mineralization in the fracture space using a grading system described by Perry et al. [29].

For the assessment of bone histomorphometry, the rabbits were sacrificed 30 days after microwave irradiation, and the bone tissue around the bone defect was obtained, and the bone samples were trimmed and fixed in 10% formalin for 1 week. After being rinsed with tap water, the samples were dehydrated with 70%-99% ethanol for 24 hours, respectively, and then embedded with methyl methacrylate. The polymerized tissue samples were placed in a refrigerator at 4°C for about one week, and the embedded samples were taken out. Sections were cut into 50 *μ*m using a hard tissue microtome and stained with toluidine blue, observed by 25x optical microscope. Measurement of the fracture gap was performed by the histomorphometry software (Simple-PCI 6.0, Hamamatsu Photonics, Japan). Nomenclature and calculations for bone histomorphometry were performed in accordance with the American Association for Bone and Mineral Research reporting specifications.

### 2.7. Ethics

All experimental procedures involving animals were performed according to the protocol approved by the Animal Welfare and Ethics Committee of Shanghai Sixth People's Hospital (License No.: SYXK (HU) 2011-0128). NIH guidelines for the care and safety of laboratory animals were strictly followed. The animals were given food and water free of charge throughout the study. All procedures and measurements were performed under deep anesthesia with sodium pentobarbital (30 mg/kg).

### 2.8. Statistics

For quantitative data, the SPSS20.0 software (IBM Corporation, Armonk, NY) was used to perform unpaired *t* test and paired sample *t* test according to the situation. Two-tailed *P* < 0.05 was considered statistically significant between the two groups.

## 3. Result

### 3.1. Temperature Changes of Tissue Adjacent to Implant during Microwave Irradiation

**Figure 4:**
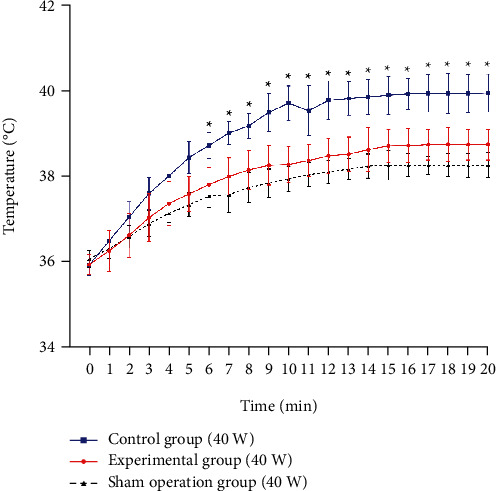
Temperature changes of deep tissues under 40 W microwave irradiation. The initial temperature of the three groups was basically the same. The body temperature of the three groups increased rapidly in the first 10 minutes and remained stable in the last 6 minutes. There was no significant difference between the experimental group and the sham operation group (*P* = 0.21). ^∗^There was a significant difference in temperature between the control group and the experimental group (*P* < 0.05).

**Figure 5:**
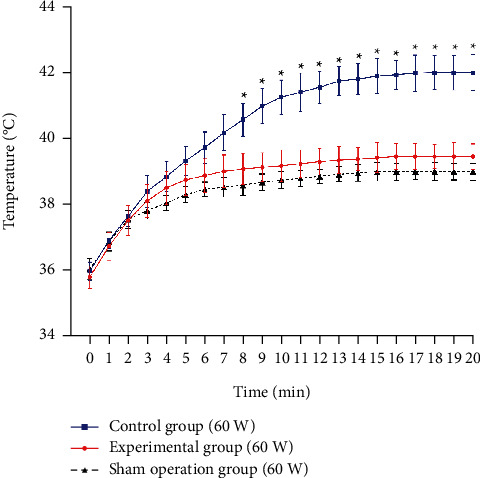
Temperature changes of deep tissues under 60 W microwave irradiation. The initial temperatures of the three groups are basically the same. The body temperature of the experimental group and the sham operation group tended to be stable after 6 minutes, while the body temperature of the control group continued to rise for about 15 minutes and then reached the peak. There was no significant difference between the experimental group and the sham operation group (*P* = 0.32). ^∗^There was a significant difference between the control group and the experimental group (*P* < 0.05).

When the microwave was irradiated at 40 W and 60 W, respectively, the temperature of the EG, the CG, and the SOG increased significantly (from 2.18 ± 0.15°C to 6.02 ± 0.38°C) (Table 1). Compared with the 40 W microwave groups, the temperature of the EG, the CG, and the SOG exposed to 60 W microwave increased more, and the average peak temperature differences were 0.72°C, 2.06°C, and 0.74°C, respectively (Table 1). When the microwave power was 40 W, the temperature of the CG rose by 4.0 ± 0.34°C, which was significantly higher than that of the EG 2.82 ± 0.15°C (*P* < 0.01) and SOG 2.18 ± 0.33°C (*P* < 0.01), and there was no significant difference in temperature rise between the EG and SOG (*P* = 0.21) (Figure 4). When exposed to 60 W microwave, the temperature rise in the CG was 6.02 ± 0.38°C, which was significantly higher than that in the EG 3.66 ± 0.14°C (*P* < 0.01) and the SOG 2.96 ± 0.22°C (*P* < 0.01), and there was no significant difference between the EG and the SOG (*P* = 0.32) (Figure 5).

### 3.2. X-Ray Assessment of Fracture Healing after Continuous Microwave Irradiation

**Figure 6:**
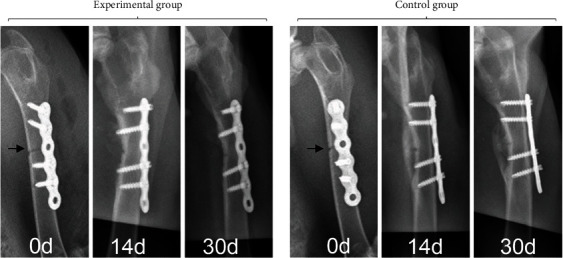
Radiographic images of femoral defects in rabbits at 0, 14, and 30 days. After 30 days, the fracture defects in the two groups were basically healed, and the fracture line was not obvious. Arrows: fracture line.

**Figure 7:**
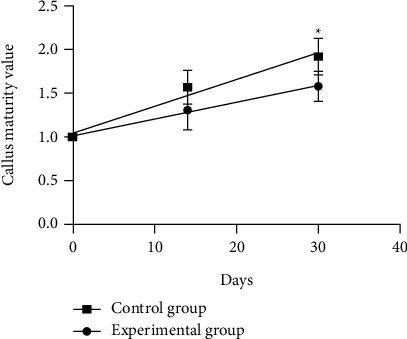
X-ray evaluation of fracture healing. ^∗^The maturity of callus in the control group was higher than that in the experimental group (*P* < 0.05).

There were significant differences in fracture line density between the EG and the CG at 0, 14, and 30 days (Figure 6), indicating that the fracture healing process of the two groups was normal. The results of callus maturity measurement showed that there was no significant difference in callus maturity between the EG and the CG at 14 days (*P* = 0.554), and the callus maturity of the EG was less than that of the CG at 30 days (*P* = 0.041) (Figure 7).

### 3.3. Analysis of Bone Histologic and Histomorphometric Data after Continuous Microwave Irradiation

**Figure 8:**
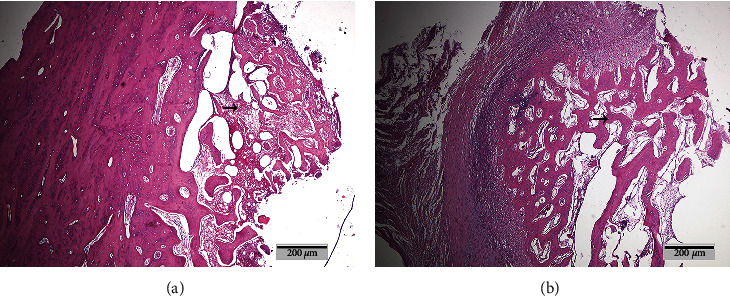
Histological evaluation of fracture healing. (a) Experimental group and (b) control group; →: bone trabecular.

Bone histopathological section showed that after 30 days of microwave irradiation, bone defects in both groups healed well, periosteal reaction was obvious, and callus proliferation was obvious (Figure 8). The fracture healing histologic score was 7.00 ± 0.76 in the CG and 6.12 ± 0.64 in the EG, with a statistically significant difference (*P* = 0.026).

The results of fracture healing histologic score and histomorphology after 30 days of microwave irradiation are shown in Table 2. The histologic score, bone volume, and node-terminus ratio of the CG were higher than those of the EG, and the differences were statistically significant (*P* < 0.05).

## 4. Discussion

In this study, it was found in vivo that TiO_2_ nanofilm could reduce the heat generation of titanium alloy implants after microwave irradiation, and it was observed that TiO_2_ nanofilm had no adverse effects on fracture healing, so it was in line with the original design.

### 4.1. Heat Production Mechanism of Metallic Implant in Microwave Field

In rehabilitation medicine, microwave therapy is a common treatment for acute injuries and chronic inflammation of bones [7–9], joints [10], skeletal muscles, and tendons [11, 12]. One of the contraindications of microwave therapy is that it cannot be applied to local metal implants [13]. There are two reasons for this. Firstly, the metal will reflect microwaves, resulting in secondary irradiation to the tissue at the treatment site [13]. Secondly, as a magnetic substance, metal generates heat in the microwave field [30]. At present, the mechanisms of heat production of metal implants in microwave fields include eddy currents and magnetic hysteresis [31–33]. Eddy current refers to the induced electromotive force generated by metal magnetic materials in the alternating magnetic field, which generates a circular current in the direction of the change of magnetic flux. Eddy currents convert the energy of electromagnetic waves into thermal energy within the metal conductor, thereby raising the temperature of the metal, that is, eddy current loss [34]. Hysteresis is a phenomenon that the change of magnetization or magnetic induction of ferromagnetic substance always lags behind the change of magnetic field intensity in the process of magnetization and demagnetization of ferromagnetic substance. Hysteresis loss is the decay and loss of energy induced by hysteresis. It is generally expressed in the form of thermal energy [35]. Hysteresis loss is related to the number of magnetic domains in the metal, and the difference between different metals is large.

In this study, titanium alloy implants were coated. The main reason is that the magnitude of eddy current loss is related to factors such as the way the magnetic field changes, the movement of the conductor, the geometry of the conductor, and the permeability and conductivity of the conductor [36, 37]. Compared with stainless steel, copper, nickel, and other metals, under the same conditions, titanium alloy has the lowest eddy current loss and heat production because of its low permeability and conductivity. In addition, titanium is a paramagnetic substance [38], with a small number of magnetic domains and less heat production due to hysteresis loss.

### 4.2. Influence Factors of Tissues Temperature Adjacent to Metal Implants in Microwave Field

It is obvious that the heat production of metal implants in the microwave field is the main factor for the temperature rise of adjacent tissues, but there are other important factors that can affect the temperature of tissues, including the dose of microwave, the duration of microwave irradiation, the distance between microwave probe and tissue, local blood flow, local tissue structure, and properties [3, 13]. The dose and duration of microwave irradiation are directly proportional to the tissue temperature, that is, the greater the microwave dose, the longer the irradiation time, and the higher the tissue temperature. The distance between microwave and tissue and the abundance of blood flow are inversely proportional to tissue temperature, that is, the larger the distance between microwave probe and tissue, the richer the local blood flow, the faster the flow rate, and the slower the rise of tissue temperature. The structure of tissues also affects the absorption and heat production of microwaves. Generally speaking, the temperature at the junction of different tissues increases due to the reflection and refraction of microwaves, and tissues with high dielectric constant absorb more microwaves, such as muscles, liver, and kidney [3]. The final temperature of the tissue adjacent to the metal implant in the microwave field is often the result of the combined effect of the above factors.

In addition, it should be noted that for metal implants in the microwave field, the temperature of the area around the implant is not exactly uniform. It has been shown that the temperature at both ends of the metal implant in the microwave electric field is the highest [39]. In this study, preliminary tests showed that there was no significant difference in temperature between the two ends of the metal implant and the central area, and that the distance from the implant was 5 mm compared with 10 mm. The temperature is higher at 5 mm [30], so we placed the temperature probe at 5 mm directly above the middle hole of the titanium alloy implant to obtain the most accurate temperature in this area.

### 4.3. Reduction of Heat Production of Titanium Alloy in Microwave Field by TiO_2_ Nanofilm and Its Possible Mechanism

The results of this study showed that under 40 W and 60 W microwave irradiation, the temperature of the CG with traditional titanium alloy plate increased more than that of the EG (*P* < 0.01) and the SOG (*P* < 0.01), while there was no significant difference between the EG and the SOG (*P* > 0.05). This indicates that the TiO_2_ nanofilm can obviously reduce the heat generated by the coated titanium alloy or/and prevent the heat generated by the titanium alloy from conducting outwards. Even if the microwave power increases, it can also play a role in blocking heat. We speculate that the mechanism may be related to the properties of TiO_2_. TiO_2_ is a semiconductor, and the TiO_2_ film is used to protect against electromagnetic irradiation [40]. When the titanium alloy coated with TiO_2_ nanofilm receives electromagnetic irradiation, its resistance is less than that of the muscle tissue and greater than that of the titanium alloy substrate. It is both a physical barrier and an electrostatic barrier at the interface between titanium alloy and muscle tissue. Therefore, it can not only reduce the occurrence of eddy current in titanium alloy but also prevent the heat generated by titanium alloy from being transmitted outside [27].

### 4.4. Effect of TiO_2_ Nanofilm on Fracture Healing

Some studies have achieved antibacterial and accelerated fracture healing by surface modification of internal fixation materials [41]. Hu et al. [42] prepared ZnO-TiO_2_ coating by microarc oxidation and compared it with pure titanium, and the short-term function (adhesion, proliferation, and alkaline phosphatase activity) and long-term differentiation of rat bone marrow stem cells (bMSCs) on zinc-containing coating were significantly enhanced. In another study [43], Zn-doped TiO_2_ nanotubes can not only inhibit the growth of bacteria but also promote the growth of osteoblasts in vitro and bone formation in vivo. There are also some recent studies showing that the addition of Fe [44] and Cu [45] elements to TiO_2_ coatings is a potential approach that can improve the antibacterial and osteogenic properties of orthopaedic implants. But in this study, TiO_2_ is a smooth and thin TiO_2_ nanofilm coated on a titanium alloy (Ti6Al4V) substrate by a sol-gel method, and the effect of increasing fracture healing is not considered in the design [27]. Therefore, this study did not observe that the fracture healing of the EG was better than that of the CG. This improvement can be considered in the future.

In the present study, more callus was observed in the CG than in the EG, and we considered that it might be related to temperature. The healing process of fracture can be influenced by the local tissue temperature [46]. A moderate increase in local tissue temperature (generally considered to be less than 45°C [3]) accelerates blood flow [13], accelerates tissue metabolism [3], and promotes osteoblast differentiation [46]. Under 40 W microwave irradiation, the temperature of the CG was always higher than that of the EG, so the CG produced more callus.

### 4.5. Defects of the Research

Firstly, in the thermometry experiment, skeletal muscle and nerve specimens were not taken to observe histological changes, so the thermal injury caused by temperature rise could not be observed. Secondly, in the experiment of observing fracture healing, limited by the number of plates, we did not set up a blank control group (that is, no microwave irradiation treatment after fracture internal fixation) and a 60 W microwave irradiation group, so we cannot judge the accurate effect of TiO_2_ film on fracture healing under different doses of microwave. Thirdly, the temperature rise of the metal implant under microwave irradiation is affected by the shape, thickness, and other factors of the implant and is limited by the size of the rabbit femur. This study only explores the heat production of the line titanium alloy plate, and the research design can be further optimized in the future.

### 4.6. Implications of the Research

The progress of material science, especially the wide application of titanium alloy implants, makes it possible to apply low-dose microwave therapy (≤40 W) to patients with titanium alloy implants, such as fracture patients with titanium alloy internal fixation [24, 30]. In this study, titanium alloy implants were coated with TiO_2_ thin film to reduce the heat generation during microwave irradiation, so that the application dose of microwave therapy was increased (≤60 W). This study opens up a promising new idea that the contraindication of local metal implants in microwave therapy may be eliminated through further improvement of material science, so that more patients can benefit from electrotherapy.

## 5. Conclusion

Under the animal experimental condition, compared with the common titanium alloy implant, the TiO_2_ nanofilm can reduce the heat production of the titanium alloy implant in a 2450 MHz microwave field and has no adverse effect on fracture healing. This study is a promising new method to increase the dose of microwave applied to titanium alloy implants by improving the surface properties of metal implants. However, the thermal insulation mechanism and dose-effect relationship of TiO_2_ film in microwave field are still not clear, whether TiO_2_ film is still effective after changing the shape, size, and thickness of titanium alloy internal fixation and whether there are other better thermal insulation coatings need more research.

## Figures and Tables

**Table 1:** Peak temperature changes of deep tissue during microwave irradiation.

Power	Groups	Temperature (*x̅*±*s*) (°C)			*P* value
		0 min	20 min	Gap	
40 W	Experimental group	35.95 ± 0.24	38.74 ± 0.36	2.82 ± 0.15	<0.01^∗^
	Control group	35.94 ± 0.24	39.94 ± 0.44	4.00 ± 0.34^#^	<0.01^∗^
	Sham operation group	36.08 ± 0.20	38.26 ± 0.29	2.18 ± 0.33	<0.01^∗^
60 W	Experimental group	35.80 ± 0.34	39.46 ± 0.38	3.66 ± 0.14	<0.01^∗^
	Control group	35.98 ± 0.28	42.00 ± 0.55	6.02 ± 0.38^#^	<0.01^∗^
	Sham operation group	36.04 ± 0.32	39.00 ± 0.26	2.96 ± 0.22	<0.01^∗^

^∗^Tissue temperature after 20 minutes was statistically different from that initial temperature (*P* < 0.01). ^#^There was significant difference between the control group and the other two groups (*P* < 0.05).

**Table 2:** Comparison of the histologic and histomorphometric data.

	Experimental group	Control group	*P* value
Histologic score	6.12 ± 0.64	7.00 ± 0.76	0.026^∗^
Bone volume (%)	22.45 ± 8.69	32.73 ± 5.86	0.015^∗^
Trabecular thickness (mcm)	19.57 ± 2.32	21.14 ± 2.45	0.082
Trabecular separation (*μ*m)	16.24 ± 4.15	14.76 ± 2.61	0.252
Node-terminus ratio(/mm)	3.25 ± 1.03	4.50 ± 0.93	0.023^∗^

^∗^After 30 d microwave treatment, compared with the experimental group, the histologic score, bone volume, and node-terminus ratio of the control group were significantly higher (*P* < 0.05).

## Data Availability

The study data presented may be made available from the corresponding author upon reasonable request.
